# Older Chinese migrants’ experiences of remote primary care in England: a semi-structured interview study

**DOI:** 10.3399/BJGPO.2025.0106

**Published:** 2026-02-25

**Authors:** Haoyue Guo, Rachael Frost, Greta Rait, Fiona Burns

**Affiliations:** 1 Institute for Global Health, University College London, London, UK; 2 Public Health Institute, Liverpool John Moores University, Liverpool, UK; 3 Research Department of Primary Care and Population Health, University College London, London, UK

**Keywords:** aged, digital health, health services accessibility, primary health care, migrants, general practice, qualitative research

## Abstract

**Background:**

Over the past decade, remote (non-face-to-face) services — including interactions via the telephone and online platforms — have been increasingly used in primary care. These services bring potential benefits, as well as potential barriers, for patients. Older migrants are a population that could face intersectional barriers when accessing health care; it is important to understand the impact of remote services on them.

**Aim:**

This study explores older Chinese migrants’ experiences of, and attitudes to, remote access to primary care services.

**Design & setting:**

A qualitative semi-structured interview study.

**Method:**

Recruitment was carried out in 2023, through community organisations, social media, and snowballing. Participants were individuals aged ≥60 years, who self-identified ethnically as Chinese, and were UK residents; they were purposively sampled for maximum variation in sociodemographics and backgrounds. Data were collected through semi-structured interviews conducted in English and Mandarin. Interviews were recorded and transcribed verbatim; if consent to record the interview had not been given, field notes were taken. Transcripts and field notes were analysed using reflexive thematic analysis. Results were shared with participants for verification.

**Results:**

Nineteen participants were interviewed. Many technical and practical barriers were reported as existing for the participants when accessing primary care remotely. Due to the different levels of access to resources, these barriers affected the most disadvantaged people to the greatest degree. In addition, participants felt the need for in-person interactions to address some concerns and believed remote services should not replace in-person care.

**Conclusion:**

Overall, older Chinese migrants felt few benefits from using remote primary care services. In the current digital context of the NHS, it is crucial to keep multimodal services available while rolling out new service modes, and to consider the needs of different populations to ensure equitable access.

## How this fits in

Remote services provide a flexible alternative route of access to primary care, but also pose challenges in ensuring equity in terms of service access and quality. This study explored the perspectives of older Chinese migrants — a potentially disadvantaged population in terms of healthcare access — on using remote primary care. The results suggest this population can face many challenges when using remote services; these relate to communication and technical barriers, as well as emotional needs being unmet during medical interactions. Service providers should ensure face-to-face services remain available, and minoritised populations’ specific needs (for example, interpretation services) are both considered and incorporated into service design.

## Introduction

Remote services — in which the patient and healthcare professional interact without physically meeting — are increasingly common in health care.^1,2^ Remote interactions could happen through a number of channels, including telephone, mobile applications (apps), text messages, and websites. Many of these interactions involve the use of the internet and smart devices (for example, smartphones and tablet computers), but could also happen purely via the telephone. They are not limited to consultations, and include other forms of healthcare access, such as appointment making and prescription management. The COVID-19 pandemic prompted an acceleration in the use of such methods in the UK.^3^ In 2020, 39.0% of NHS primary care appointments in England were carried out via telephone or video calls; in 2024, telephone and video consultations still constituted a significant proportion of primary care appointments in England at 30.6%.^4^

Remote services are thought to remove practical barriers, such as transportation, and provide an easier environment for discussing sensitive topics.^5–10^ However, those who are less familiar with technology or have limited access to digital devices and the internet are at risk of exclusion.^11–15^ In addition, other barriers may interact with technical issues to impact remote healthcare access and outcomes;^16^ as an example, health mobile apps are considered more useful by people with higher levels of health literacy.^17^ People’s attitudes towards technology may also be affected by emotions associated with changes in healthcare delivery; a study among older rural residents in Sweden found avoidance of remote care to be a form of resistance to what they perceived as retrenchment policies.^18^

Older migrants represent a population that may face overlapping factors that influence their access to health care, such as mobility, language, and difficulties navigating the healthcare system.^19–21^ They may face additional technical and/or sensory challenges; however, they could potentially benefit from remote services by avoiding transportation and using written messages as an easier alternative to verbal communication.^22–24^

According to the 2021 census, there were >445 000 ethnically Chinese residents in England and Wales;^25^ of these, nearly 57 000 were aged ≥60 years.^26^ Residents born in China or Hong Kong totalled >300 000 in England and Wales,^27^ but Chinese migrants — especially older Chinese migrants — appear to be an understudied population in the UK. Previous research on this population has mostly been conducted in the US.^28^ Older Chinese migrants face migrant-specific difficulties, such as language barriers and issues with navigating a different healthcare system; in addition, unique cultural factors — including the use of traditional medicine and agreement with its values — are found to have an impact on healthcare access for this population in high-income countries.^20,21,29^

This study focused on older Chinese migrants to explore their experiences in their specific sociocultural context and in the rapidly changing UK healthcare system. It aimed to:

- understand older Chinese migrants’ opinions and attitudes towards remote primary care through their experiences; and
- provide insights into service improvement to ensure equitable access for this population.

## Method

### Recruitment

Participants were UK residents who:

- self-identified as ethnically Chinese;
- were aged ≥60 years; and
- had been born outside of the UK.

Participants were recruited via community organisations and social media. Snowballing — whereby participants are asked to spread word about the study to other potential participants^^30^^ — was also used. Participants were purposively sampled for maximum variation in sociodemographics and migration backgrounds. The theory of information power^31^ was employed to help estimate the sample size; based on the research topic, target population, and analytical approach, we initially estimated that 20 interviews might be needed. Recruitment stopped when all major topics of interest had been explored.

### Data collection

Data were collected through semi-structured interviews conducted in English or Mandarin in person or via telephone. The interviews were audio-recorded with the participant’s consent; when a participant did not consent to recording, field notes were taken. The interviews were conducted from December 2022 to May 2023.

A topic guide (see Supplementary Information S1) was developed based on previous literature, Levesque *et al*’s^32^ access to healthcare framework, and input from public and patient involvement activities. Key topics were:

- experiences and opinions of primary care;
- assistance during healthcare access;
- alternative medicine; and
- suggestions for services.

The topic guide was continuously modified as interviews were carried out to reflect new areas of interest that emerged.

### Data analysis

Recordings were transcribed verbatim in the interview’s original language by the interviewer, and the transcripts were analysed alongside field notes. We took a constructivist stance and used Braun and Clarke’s^33^ steps of reflexive thematic analysis as a guide for the analytical process. We explored the impact of remote services on primary care through the lens of the healthcare journey proposed by Levesque *et al* ^32^ — namely, that optimal access consists of the whole process, from realising healthcare needs to meaningful engagement in care. Although we referred to Levesque *et al*’s^32^ framework for healthcare access to define access and guide the research, we employed an inductive approach to data analysis to do a ‘deep dive’ into the sociocultural context.

The transcripts were analysed without translation by the interviewer, who is bilingual, and coded directly in English over three rounds at different orders. The codes were categorised and discussed with the other three researchers to ascertain shared concepts and themes; final themes were generated through several iterations of this process. Excerpts of the original transcripts were translated into English to aid discussion.

In addition to the themes generated, we also mapped out the participants’ accounts (as presented in the results) and the respective user or provider characteristics that impacted the healthcare access journey according to Levesque *et al*’s^32^ framework. This map provided a summary of the results from the healthcare journey perspective, and the themes highlighted their significance for participants.

A summary of the results, along with supporting quotations in both English and Mandarin, were sent to participants for checking; we did not receive any additional input or requests for change.

### Research team and reflexivity

The research team consisted of a doctoral researcher, who was the interviewer, and three senior researchers. The interviewer is female, originally from China, and speaks both Mandarin and English. The interviewer felt that the common ethnic identity helped to build rapport with research participants, especially female participants. Due to a shared migration background with the interviewer, some participants particularly shared thoughts from a migration perspective, commenting on whether NHS services make England a better or worse migration destination.

## Results

### Participant characteristics

Interviews were conducted with 19 participants; there were 13 female participants and the median age was 73 years. Participants’ characteristics are given in Table 1. Where quotations are presented, participant characteristics are summarised in brackets.

**Table 1.** Characteristics of study participants (*N* = 19)

Characteristic	*n*
**Sex**	
Female	13
Male	6
**Age, years**	
60–64	4
65–69	1
70–74	8
75–79	6
**Years in the UK**	
>40	11
20–40	6
10–19	0
5–9	1
<5	1
**Education**	
Primary school	3
Secondary school (years 7–9)	1
Secondary school (years 10–12)	6
College or above	9
**Place of birth**	
Hong Kong	6
Mainland China	4
Malaysia	7
Thailand	1
Cambodia	1
**Place of residence**	
Greater London	16
Birmingham	1
’Small towns‘ (name not given)	2
**Recruitment method**	
Community organisation	12
Snowballing	5
Social media	2
**Interview language**	
English	11
Mandarin	8
**Interview setting**	
In person	15
Telephone	4

### Theme 1: the practicality of accessing primary care through remote services

Participants described various ways they had interacted with healthcare professionals remotely, including telephone conversations, website-based forms, mobile apps, and text messages. Most participants used a combination of telephone and online services; consultations mainly happened over telephone with complementary online services (for example, online records of appointments). This theme covers participants’ general attitudes towards technology and technical skill, as well as the specific challenges in the context of healthcare access.

When asked about their thoughts on smart devices and the internet, participants agreed that they were commonly used and a part of everyday life in society; as one participant noted:

*‘Yeah, smartphone, everybody got phone nowadays, one or two normally, in the pocket, yeah?’* (Male [M][participant number]19, aged 74 years)

However, participants mentioned that some older people might still have limited access to smart devices or the internet, especially when the cost of smart devices presented an additional barrier:

*‘For a lot of people over 60, not everyone has internet all the time … like my dad, he has a landline phone upstairs … there’s no internet up there.’* (Female [F]8, aged 61 years)

*‘You know all these people, you know, like my friend’s daughter, they pay for iPhone over 1000 pounds, blimey … ’* (F5, aged 74 years)

Most participants said they were confident with frequently performed tasks, such as contact with family and friends, entertainment, and obtaining information; however, accessing health care remotely was considered a more complex task. Learning to access healthcare services through remote channels posed a challenge, which could deter people from using certain remote modalities:

*‘Technology, I think sometimes is, also very lazy to learn, I’ve got an iPad, only I used it for certain things, I'm quite happy with that ... I suppose if I wanted to I can Skype the doctor, make an appointment and see the doctor face-to-face, you know, that way I can, but sometimes is so much ... I've never tried it, never occurred to me to try.’* (F12, aged 76 years)

Answering a call from GP surgeries was not always easy. Several participants mentioned the struggle to be ready at all times, due to the uncertainty of when the surgery might call. They feared missing the call and hence missing their appointment:

*‘Then you have to wait for the doctor’s call, they cannot promise you “I will ring you in the morning” or not. You have to stay, you bring your mobile phone into the toilet, in case … ’* (F6, aged 74 years)

In addition, sensory conditions, such as declining eyesight or hearing, presented barriers to using certain devices:

*‘I don’t like using the phone, the screen is too small, ahh … when looking at the website on a computer, well maybe my eyes aren’t that good now with the age, I could see when the print is large … and the* [GP appointment] *website is designed to have you read a lot of text first.’* (M4, aged 61 years)

Despite the difficulties, the use of remote services was not completely without its merits. Participants noted that being able to access their GP remotely could benefit people who had problems travelling to the GP or who were working, although these conditions did not always apply to them:

*‘But also, it will be beneficial for people who live far away, I think. When you have problem travel to your GP, I think communication like this is excellent for them ... I’m just aware that it doesn’t convenient me, but I also see that it might be convenient for other people.’* (F5, aged 74 years)

### Theme 2: widening inequities

For older migrants who already faced challenges accessing health care — for example, due to language barriers and difficulties — remote communications exacerbated language problems:

*‘Because for a lot of Chinese they aren’t completely fluent in the language ... for locals, when they communicate, they don’t have any problem on the phone ... And because of the local accent, in that scenario, you can’t call it language problem, but an accent problem, it’s harder to understand.’* (M4, aged 61 years)

It can also be harder for non-native speakers to communicate without visual cues, as they must on the telephone, especially when technical terms may be used. One participant, who acted as an interpreter for a local Chinese community centre, mentioned that interpretation was less engaging over the phone compared with in person; this was especially the case when calls were not set up appropriately:

*‘Of course not the same* [as in person] *isn’t it, not the same in the sense that I can’t see what the situation* [is]*, the patients at the hospital, yeah, so it’s not properly arranged I suppose, if you're proper, like arranged by the hospital, I think there’s a three-way conversation, yeah, but for m*e *because it’s last-minute things* [that interpretation was not set up properly as a three-way conversation]*.’* (F16, aged 63 years)

With written English, the use of technical terms on websites and the need to write free-text answers also posed a challenge for non-native speakers:

*‘That* [online form] *feels like composition, maybe it’s easier for British people, but for foreigners, like us, although I’ve been here 30 years, some words you say more, but not for reading or writing, so you know, it feels like composing an article to me.’* (F8, aged 61 years)

In addition, many of the practical barriers mentioned in the first theme required social resources to be overcome; as such, remote services could widen inequities. Some migrants could easily mitigate difficulties by seeking help from personal or community networks, but this source of assistance was not guaranteed for all:

*‘Oh, I’m computer illiterate, everything my children does it for me, I don’t have to lift my finger.’* (F11, aged 72 years)

*‘Because some family have grandchildren to help. I live on my own, I don’t have children, I can’t, I don’t have anyone to borrow, and my neighbours, they’re so busy, they don’t offer help, you know.’* (F5, aged 74 years)

Another participant pointed out that the different forms and sizes of local communities affected available support and resources. Members of established and larger communities could access more help:

*‘We’ve been here for a long time. What about some of the new groups, you know? How are they finding the situation, and they won’t have community centres to come to … Chinese are considered quite established, yeah. Our network, they contact us and the doctor knows us, professionals know … So we are, I suppose, stronger in terms of providing support for our community ... ’* (F16, aged 63 years)

The reasons for migration and socioeconomic status added additional layers to the overlapping inequities. Migrants with a lower level of education were more likely to have less-skilled jobs and could become more dissociated from mainstream society as a result. For them, learning to adapt to new services was harder, as they had fewer resources for information and learning:

*‘I think there are two groups of people over 60: some came as elites, they were PhD, they came to work in universities, and they have no problem. Others are like me, traditional medicine, restaurants, small businesses, how are you going to learn? Who’s gonna teach you?’* (F8, aged 61 years)

### Theme 3: necessity of in-person interaction for medical concerns

Depending on the type of healthcare interaction and the individual’s perception of what was needed to fulfil its purpose, in-person interactions could be considered an integral part of care. The need for health care can vary depending on personal beliefs and preferences; these preferences sometimes relate to cultural backgrounds and an individual’s level of agreement with them. Some participants mentioned using traditional medicine for their everyday ailments, and only turning to GPs for serious concerns; in such cases, face-to-face interactions were important:

*‘I usually take herbs … and then sometimes it still not working, and that’s when I go see GP … it’s a psychology, you know, I have to see someone and talk to someone.’* (F5, aged 74 years)

Expectations for primary care also related to personal attitudes towards the internet and the authority of clinicians in health care. In contrast with participant F5, another participant said they were comfortable searching for information online and making decisions themselves; they perceived consultations as a way of obtaining referrals and preferred the simplest form of care delivery:

*‘That’s my understanding, it’s not whether I need you* [the GP]*, I know what’s going on, maybe if I spend more time searching it’s better. The role of a GP for me now is, well, I have to go through you to get a referral, after GP* [consultation]*, right? … I like, just a voice call is fine, if I want to show the GP something on me, I’d do a video call, if it’s just description then a voice call, the simpler the better.’* (M10, aged 60 years)

Most participants expressed that, when approaching healthcare professionals about new concerns, the GP was needed for care, direction, and reassurance. Trust building and personal connections were considered necessary. The role of GPs was complex and personal; they represented a figure the participant could fully rely on for reassurance. However, trust building and human connection in a GP consultation was not always realised when receiving care remotely:

*‘... other things can be remote, like buying stuff, selling food, or other things, there are no emotions involved, these things don’t involve feelings. But for doctors, I feel there is, um, there are emotions, other than feelings, there’s also the issue of trust or not trust, so I don’t know, I can’t really accept this* [remote service]*.’* (F8, aged 61 years)

An in-person visit to the GP offered more certainty of being taken seriously and cared for, in the form of non-verbal cues and physical examinations. This offered further reassurance for patients who might have felt unsure about their condition and the next steps:

*‘At least face to face they really can see your problem, like my coughing, when I talk to them at the beginning they don’t really got it, I believe they will just say, uh, “Because all this cold eventually will go away” ... I must say that when you got a cold you go in and see, then they always listen to your chest.’* (F14, aged 76 years)

Although the vast majority of participants preferred in-person consultations, participants also confirmed they would be comfortable performing repeated tasks online, such as ordering prescriptions with delivery services; this service was considered a more convenient alternative to traditional prescription management:

*‘It’s just that I, my prescription, now I do it online, because for the prescription just saves a lot of time, yeah, and then the GP will send the prescription direct to the pharmacist, then I gotta pick up my prescription, and that’s good.’* (M9, aged 77 years)

### Theme 4: replacement of face-to-face services

During the COVID-19 pandemic, remote services were used more widely because face-to-face interactions were restricted. However, after these restrictions had been lifted, some participants expected face-to-face services to return to pre-pandemic levels:

*‘I mean the spot of GP is in the surgery, with appointment we turn up and see them. COVID is nearly gone, alright. If they scared, we wear mask, yeah? They can wear mask. But let us go and see them.’* (F7, aged 72 years)

Instead of becoming an alternative option to face-to-face services, remote services remained the only option to access care for many participants. The inability to make appointments face to face was frustrating for some, who often made the journey to the practice because they had encountered difficulties in contacting the practice remotely:

*‘And one morning, I was there, I went to make an appointment, I said “Help me make an appointment, I want to see the doctor”.* [They said] *“Call tomorrow morning”. I mean, I was there in person!* [If I] *call them the next morning at 8, the call won’t go in. How can they do that? Because they tell everyone to call at 8, everyone is calling, and I am here in person, they just won’t make an appointment for me, just no, it has to be over the phone.’* (F17, aged 78 years)

Another participant worked for a local community centre and so often made GP appointments on behalf of older Chinese people who lived in the community centre’s charity housing. She was frustrated that she had to complain several times for the surgery to make adjustments:

*‘I got really upset, said “Look, these are the way I called for the past few days trying to make appointment every time”, because I waited 40 minutes, 50 minutes, I give up, you know? … I said “Maybe may I suggest we forget, I can walk up to make the appointment, maybe it’s better than me holding up the phone”.’* (F16, aged 63 years)

After making an appointment, the first contact with the GP was often a remote consultation:

*‘I think for this particular surgery, I think they always, the initial, the first time they like to give phone consultation, after that, yeah, they will decide.’* (F16, aged 63 years)

Although some participants had a smoother experience of seeing their GP face-to-face after an initial remote consultation, others encountered difficulties that made them feel they were being actively prevented from face-to-face services. For those who did not see remote services as an equal alternative to face-to-face services, this was perceived as meaning the NHS did not want them to receive health care:

*‘The best is, they hope you don’t see any GP.’* (M4, aged 61 years)

*‘Stupid, no good. After COVID they will prefer you dying than seeing you.’* (F6, aged 74 years)

Notably, the mode of consultation appeared to be, predominately, decided by the GP surgery. Few participants mentioned that their surgery would ask for their preference on consultation type while making an appointment, and what they received did not always align with their preference. Overall, they felt passive in deciding the mode of service:

*‘... how do you want to be seen, or do you have a telephone conversation, but sometimes you put “to be seen”, they’ll still put telephone conversation.’* (F11, aged 72 years)

Overall, the unavailability — or limited availability — of face-to-face services was received negatively by participants and seen by some as the NHS failing to fulfil their responsibilities.

### Remote primary care from a patient journey perspective

**Figure 1.**
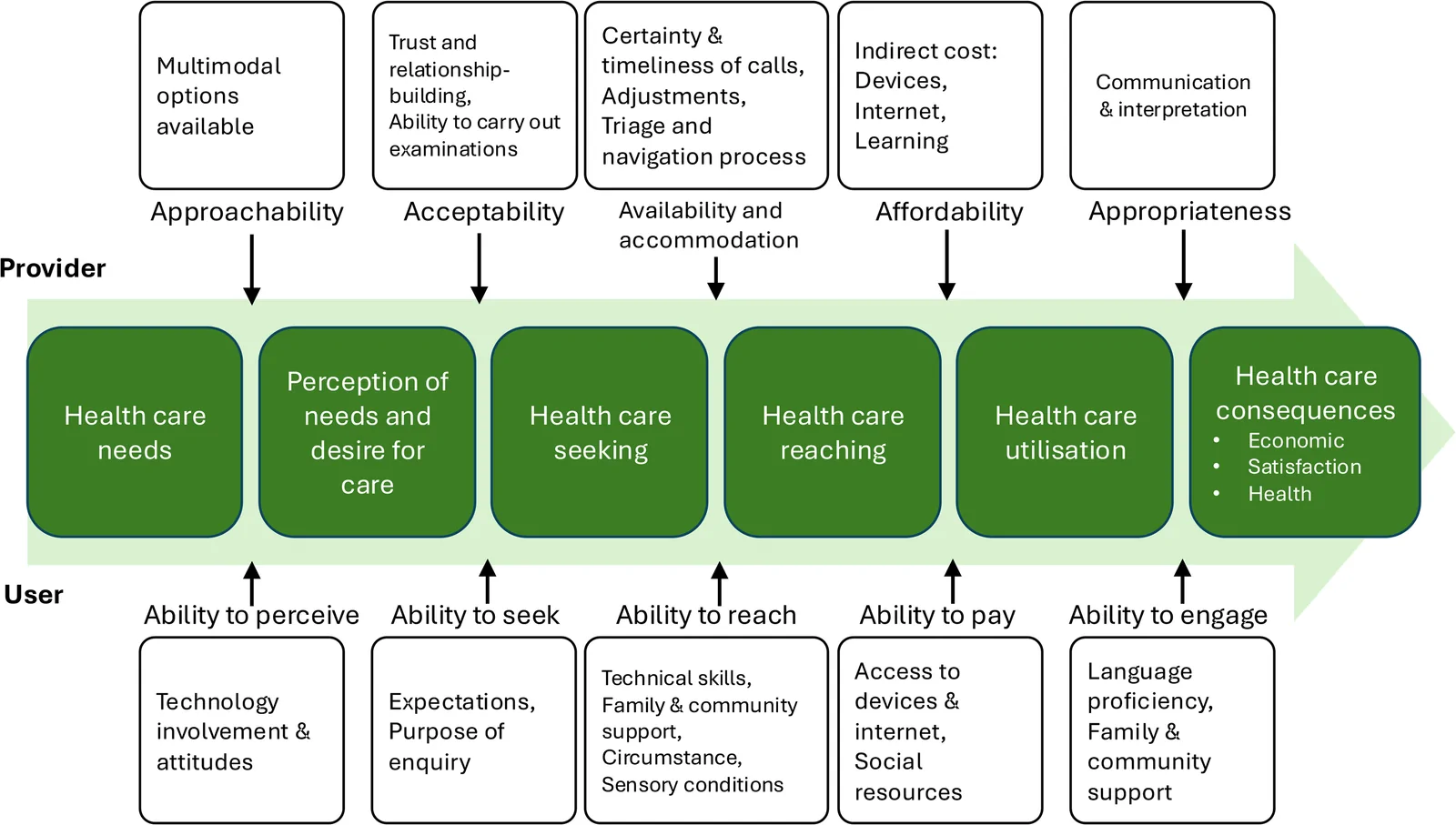
Factors influencing older Chinese migrants' use of remote primary care mapped onto Levesque *et al*’s^32^ framework

We mapped our findings onto the healthcare access journey proposed by Levesque *et al* ^32^ in order to summarise the provider and user characteristics that might influence the use of remote services for primary care; this is shown in Figure 1. A number of provider and user characteristics were shown to impact the journey of accessing remote primary care in the interviews. It was also noted that:

- people would only perceive a need to use remote services if they were aware, and held an open attitude, towards such modalities;
- remote services could be deemed unacceptable, depending on the patient–clinician relationship and the medical nature of the enquiry;
- an individual’s ability to reach the service could be influenced by their technical skills, sources of support, sensory conditions, and personal circumstances; and
- during a remote clinical interaction, linguistic barriers were a particular challenge for migrants.

Although NHS services are free at the point of delivery, several indirect costs can be generated in the process, such as the cost for internet connection or equipment.

## Discussion

### Summary

Many older Chinese migrants in our study considered remote primary care services to be a contingency solution during the COVID-19 pandemic, and most participants did not think remote services benefitted them. Practically, remote services could be difficult to navigate, and their benefits, for the most part, were not seen as being applicable to the study participants. The need for trust building and personal interactions was not always fulfilled during remote interactions. In addition, remote services were perceived as being of lesser quality compared with face-to-face interactions, and exacerbated inequities.

### Strengths and limitations

This study covered a population that has received little attention, providing a unique perspective on the role of remote services in primary care access in the UK. The recruitment took a maximum heterogeneity approach: participants were from different countries and had different levels of educational attainment, social backgrounds, migration circumstances, and English proficiency levels. However, some subgroups of the population may have been missed from the study.

Although efforts were made to reach Chinese communities outside of London, most participants lived in or near London; as a result, most experiences were from urban areas. In addition, London has a large overall migrant population,^34^ with more community resources and better support for migrants from diverse backgrounds, so the findings of this study are not as transferable to minoritised ethnic groups or to migrants in non-urban settings. Most participants had been in the UK for ≥20 years, and there were limited data to explore the experiences of those who had migrated to the UK very recently.

None of the participants required assistance for mobility and none spoke about having physical difficulties in visiting the GP, so there was no opportunity to explore how someone with mobility issues might feel about being able to access care without travelling.

The interviews were conducted in Mandarin and English; although there was an option to use an interpreter, no interviews were conducted with Cantonese-only speakers. Given the widespread use of Mandarin in Chinese ethnic migrant groups (such as those from mainland China, Taiwan, Malaysia, and so forth),^35^ Cantonese-only speakers are often early migrants from Hong Kong, and represent a potentially more marginalised group.^36^

### Comparison with existing literature

Many of the concerns and challenges with remote primary care mentioned in this study have been shared by older adults and other migrant populations elsewhere: studies have shown that remote consultations are favoured by non-migrants, more-affluent and educated individuals, and working-aged people; older people may be more likely to encounter technical difficulties.^37–42^ This study identified concerns for a more transactional patient–physician relationship in a remote environment from the service users’ perspective; other studies found similar concerns among both service providers and users.^13,43,44^ A survey with a non-age-restricted sample (aged 17–97 years) in the UK showed that people thought remote primary care was convenient, but could generate additional anxiety due to the lack of in-person communication and physical examination.^45^ In general, remote communications have seemed to work better with established relationships and for minor issues;^22,24,43,46,47^ the latter point aligns with the findings of this study.

Language is a common barrier for non-native speakers that is potentially exacerbated by remote services, and was shown to apply to the participants of this study. In alignment with this, other studies undertaken in France^48^ and the UK^49^ have also found that non-native-speaking patients can face multiple difficulties in accessing primary care due to language barriers. Studies have found that, although remote interpretation can be an accessible solution, careful planning and organisation is required to deliver these benefits;^48,50^ this aligns with the findings of the present study.

Studies involving Chinese migrants have often focused on traditional values, cultural preferences, and the use of traditional medicine.^20,28,29^ Findings from the present study reflect some of these elements — for example, the use of family assistance and traditional medicine — however, there was considerable variability in attitudes towards cultural concepts among participants.

### Implications for research and practice

Difficulties in navigating remote services and accessing face-to-face care have been shown to be frustrating for many older Chinese migrants. Given the variation in digital literacy, device and internet access, language proficiency, and preferences, it is crucial to maintain the availability of face-to-face access and consider which modalities are most appropriate for which circumstances. GP surgeries should present multiple channels to accommodate different choices; it should be noted that surgeries that provide reasonable adjustments and adhere to timely delivery of services could well make access to care easier for patients.

The difficulties our study participants experienced when navigating remote services may well apply to other migrant groups, older people, and marginalised communities. However, although it is important to acknowledge ethno-cultural factors for migrants, it is equally important to avoid harmful stereotypes and alienation of migrants in health care and health research. Our findings highlight the importance of understanding service users’ experiences and needs during healthcare service design. Service providers need to acknowledge and find ways to accommodate the needs of different populations to ensure equitable care. As an example, it has been acknowledged that language is a particular challenge for migrants who speak English as a second language;^51^ policymakers and healthcare providers need to evaluate the current framework for interpretation services and ensure the availability of interpretation services for both remote and face-to-face access.

Future participatory research that engages under-represented groups, such as migrants and minoritised ethnic groups, will be useful for optimising the delivery of inclusive services in a digital era.
